# The Chattahoochee Valley Medical and Surgical Association

**Published:** 1912-07

**Authors:** J. S. Todd

**Affiliations:** Atlanta, Ga.


### THE CHATTAHOOCHEE VALLEY MEDICAL AND SURGICAL SOCIETY.

Gentlemen of

It is with great pleasure that I welcome to Atlanta, the Gate City of the South, and Queen City of this Valley; I wish each and every one of you long life, if it be devoted to the cause of humanity; prosperity, if it does not puff you up; wealth, if you use it aright; if you wish anything else I wish that too.

The Fulton County Medical Society is an offspring of the old Atlanta Academy of Medicine, organized in the sixties, it is, therefore, one of, if not the oldest local Medical Associations in the state, it has a history full of honor and usefulness. I like the name Academy of Medicine better than Fulton County Medical Society, but the name of your association was an inspiration born very probably in the loving heart of the elder Dr. Love, father of the Love who so lovingly nourishes it now.

I thank God that I was born in the Chattahoochee Valley, so close to the banks of the broad river at West Point, that I could hear the roaring of the water as it fell over the shoals at night. The river was always mysterious to me, as a child, and filled me with an awe, fully as great as was ever felt by the Hindoo for his sacred Ganges, and I worshipped its muddy waters as did the Roman the tawny Tiber. The river was used as a guide by the migratory birds as they journeyed, and many a time at night have I laid and listened to the cranes, and thought the sky was peopled with angels, when I heard the discordant note of wild geese, I imagined they were spirits gone astray.

As a youth I stood upon its banks in Alabama and looked across at the red hills of Georgia, on one of which was a female college. It appeared an immeasurable distance away to me. I would gaze until I imagined that I not only heard but saw the Goddess in the Temple calling me over to partake of her knowledge, "which was more precious than rubies, more to be desired than gold, yea, than much fine gold."

The Chattahoochee Valley contains an area of about 20,000 square miles, and is the most wonderfully beautifuly and varied of any similar one on the western hemisphere. It is 550 miles from Appalachicola, its mouth, to where it rises in sight of Screamer, Bald and Yonah mountains, in Rabun county, where Dr. Miller imbided eloquence and Judge Blakely in "solitudes was so often found listening to rainbows and looking at sounds." The valley includes the Flint, a tributary, and territory in Alabama and Florida as well as in Georgia.

Sidney Lanier says of it in his Song of the Chattahoochee:

Out of the hills of Habersham,
Down the valleys of Hall,
I hurry amain to reach the plain,
Run the rapid and leap the fall,
Split at the rock and together again,
Accept my bed, or narrow or wide,
And flee from folly on every side
With a lover's pain to attain the plain
Far from the hills of Habersham,
Far from the valleys of Hall.

All down the hills of Habersham,
All through the valleys of Hall,
The rushes cried, "Abide, Abide;"
The willful water weeds held me thrall,
The laving laurel turned my tide,
The ferns and fondling grass said, "Stay,"
The dewberry for to work delay,
And the litle weeds sighed, "Abide, Abide,
Here in the hills of Habersham,
Here in the valleys of Hall."

High over the hills of Habersham,
Veiling the valleys of Hall,
The hickory told me manifold
Fair tales of shade; the poplar tall
Wrought me her shadowy self to hold;
The chestnut, the oak, the walnut, the pine,
Overleaning, with flickering meaning and sign,
Said, "Pass not, so cold, these manifold
Deep shades of the hills of Habersham,
These glades in the valley of Hall."

And oft in the hills of Habersham,
And oft in the valleys of Hall,
The white quartz shone, and the smooth brook-stone

Did bar me passage of friendly brawl,
And many a luminous jewel lone—
Crystals clear or a cloud with mist,
Ruby, garnet and amethyst—
Made lures with a light of streaming stone
In the clefts of the hills of Habersham,
In the beds of the valleys of Hall.

But, Oh! not the hills of Habersham,
And Oh, not the valleys of Hall
Avail: I am fain for to water the plain.
Downward the voices of duty call—
Downward, to toil and to be mixed with the main,
The dry fields burn, and the mills are to turn,
And a myriad flowers mortally yearn,
And the lordly main from beyond the plain
Calls o'er the hills of Habersham,
Calls through the valleys of Hall.

I say this is a wonderful, an unique valley, and I mean what I say, and only say what is meant, when I assert that there is not a production that grows in the soil of the Untied States from Maine to California, from the Lakes to the Gulf, but what has been successfully produced in its borders. There is a precinct in Hall County called Canada, where frost occurs every month in the year. From its course 4,000 feet above the level of the sea, every variety of tree and climate can be found. For fifty miles along its source, the rhododendron, the laurel and azalea in countless variety grow to perfection, there the fir tree recalls Maine and Minnesota, poplar trees that would make a Scotchman envious lift their heads from the coves to catch the sunshine, there the walnut and the cherry grow in profusion, all the way down the wide spreading oaks remind you of England, and the water oaks of the Spanish Main. Lower down in its course as the river broadens, the air is fragrant with the odor of the yellow jasmine, and you are for 150 miles in the land "where the tall pines rise to the bending skies and the wild magnolia blossoms." The eagle has its eryie at its source, pelicans gorge themselves with fish where it loses itself in the waters of the Gulf, and alligators bask in the sunshine.

In the counties of Forsyth, Habersham, Hall, White and Lumpkin, the gold ores are four and one-half times richer in that metal than the famous Rand mines in Africa, enough of them to pay the national debt, awaiting the hand of some discoverer that will free them from their embrace with sulphur.

In 150 miles of its headlong and precipitous course there is water power enough to turn all the wheels of the machinery in the entire South to-day. The time is not far distant that when harnessed there will be enough power utilized not only to do this, but to light up the entire valley from beginning to end, from north to south, from east to west, and then "there will be no night there." If this valley were walled in, it could within itself produce every necessity and luxury that we now enjoy, save a few probably, of the tropical fruits; we might have to do without coffee for a year, but soon tea which thrives so well in this soil would take its place.

We are surrounded by "acres of diamonds" and no man fortunate enough to live here need to envy the citizens of any other part of the earth, for we have a heritage in this valley that, before the Millennium, will support ten millions of people in affluence and plenty. Verily, we live in a land "where every prospect pleases" and men are not vile, for a nobler, braver race of men, fairer or more virtuous women, or kindlier negroes, do not live. In sight of from where I stand, are buried in the national cemetery eleven thousand federal soldiers, gruesome evidences of the "bloody hands" with which the men of this valley "welcomed to hospitable graves" invaders of their land. Before the white men took possession of it, the northern portion was the home of the Cherokee Indians, who were by all odds the first among the aborigines physically, mentally and morally; so much for environment. There is not a healthier spot by nature in all this world than this Chattahoochee Valley. The northern half of it can be kept and will be kept, free from mosquitoes, and in the southern portion they can be easily exterminated; the hookworm can and will be eradicated, if we will do our duty and teach the people how not to pollute the soil and waters; the same of flies. Let us impress the people that they fight the breeding places of these causes of disease.

If we would, as we could, only raise our own corn, there would be no more pellagra. Our consumptives and pleasure seekers need not leave its borders for change of climate, winter or summer.

The following precious stones abound in the Chattahoochee Valley: ruby, opal, zircon, chalcedony, agate, emerald, amethyst, jasper, garnet, beryl and diamonds; there are mountains of marble and granite everywhere, so you see that we have nearly all the materials that are mentioned that St. John mentioned in the foundations of the "New Jerusalem," even to the gold to pave its streets, and in the waters of Appalachicola Bay we find the oysters for the pearly gates.

But a few words as to the Fulton County Medical Society, whose guests you are on this occasion. As I said before, it was organized in the sixties by such men as the Westmorelands, Logans, Rays, Armstrongs, Okeefs and Borings, all earnest, patriotic, ethical and high-toned physicians, who emerging from the poverty of the Civil War, laid the foundation for a structure greater than they ever dreamed of. When I think of these men and what they accomplished, and how they strove, the discouragements that they overcame, I am reminded of the old negro woman who, when she was told that Solomon had 700 wives, and 300 comabies, said, "Glory to God, men was men in dem days."

I have had every office within the gift of the Georgia Medical Association, and held high commission in the American Medical Association, but the one of which I am most proud, is that I was once a President of the Old Academy of Medicine. How could I fail to feel pride in having occupied a position once filled by such a man as that king among men, A. W. Calhoun, philanthropist and societiest, whose greatness and goodness will only be unfolded in its entiety, when the Judgment books are opened.

This Society at present, gentlemen, numbers among its members 90% of all the regular physicians in the city; indeed, it is a shame for a doctor to reside here and not belong to it. You will find among its enrollment scientists of the highest order, members who can make you an opsonic index, give an X-ray picture, supply you with a Wassermann or Vidal reaction, or furnish you a chemical analysis, quantitative or qualitative; surgeons who perform every operation known to the art, specialists who can diagnose any disease that flesh is heir to.

On account of there being two first-class medical colleges here, the spirit of commendable rivalry, free of envy and detraction keeps them ever alert and diligent.

As the oldest member of the Fulton County Medical Society, knowing that in the very nature of things, the day of my departure is near at hand, I leave, surrendering the proud flag of medicine to the keeping of young men, in whom I have every confidence, of whom I am proud, and all of whom I love, believing that they will never allow it to trail in the dust; in the name of this goodly company I again welcome you.

J. S. Todd.

